# Caregiver Burden Is Associated With Dementia Patient Cognitive Performance in NHATS

**DOI:** 10.1093/geroni/igab046.1686

**Published:** 2021-12-17

**Authors:** Alexandra Wennberg, Loretta Anderson

**Affiliations:** 1 Karolinska Institutet, Stockholm, Stockholms Lan, Sweden; 2 University of Maryland, Baltimore, School of Medicine, Reisterstown, Maryland, United States

## Abstract

Informal caregivers of dementia patients engage in multicomponent care that is often stressful. In heart failure patients, caregiver burden has been associated with occurrence of cardiovascular events. However, little is known about how caregiver burden affects patient cognition in dementia care dyads. Using data from the National Health and Aging Trends Study and National Study of Caregiving, we examined the association between caregiver burden, assessed on 38 aspects of caring, and patient cognition, assessed with the immediate and delay word recall, Clock Drawing, and self-rated memory. In fully adjusted models at round 7 (2017) higher caregiver burden was cross-sectionally associated with lower immediate (B=-0.02, 95% CI -0.03, -0.01) and delayed (B=-0.03, 95% CI -0.04, -0.02) word recall. Longitudinally, across rounds 7-9 (2017-2019) higher burden was associated with lower patient Clock Draw score (B=-0.01, 95% CI -0.03, -0.001). These findings have implications for economic assistance and interventions in dementia care dyad.

